# A mouse model for Luminal epithelial like ER positive subtype of human breast cancer

**DOI:** 10.1186/1471-2407-7-180

**Published:** 2007-09-20

**Authors:** MJ Mahesh Kumar, KS Ponvijay, R Nandhini, RS Nagarajan, J Jose, G Srinivas, P Nagarajan, R Venkatesan, Kishor Kumar, S Singh

**Affiliations:** 1 Centre For Cellular and Molecular Biology, Uppal Road, Hyderabad 500 007, India; 2 National Institute of Immunology, New Delhi 110067, India

## Abstract

**Background:**

Generation of novel spontaneous ER positive mammary tumor animal model from heterozygous NIH nude mice.

**Methods:**

Using brother-sister mating with pedigree expansion system, we derived a colony of heterozygous breeding females showing ER-Positive tumors around the age of 6 months. Complete blood picture, differential leukocyte count, and serum levels of Estrogen, Alanine amino transferase (SGPT), Aspartate amino transferase (SGOT), total protein and albumin were estimated. Aspiration biopsies and microbiology were carried out. Gross pathology of the tumors and their metastatic potential were assessed. The tumors were excised and further characterized using histopathology, cytology, electron microscopy (EM), molecular markers and Mouse mammary Tumor Virus – Long Terminal Repeats (MMTV LTR) specific RT-PCR.

**Results:**

The tumors originated from 2^nd^or 5^th^or both the mammary glands and were multi-nodulated with variable central necrosis accompanied with an accumulation of inflammatory exudate. Significant increases in estrogen, SGPT, SGOT and neutrophils levels were noticed. Histopathologically, invasive nodular masses of pleomorphic tubular neoplastic epithelial cells invaded fibro-vascular stroma, adjacent dermis and subcutaneous tissue. Metastatic spread through hematogenous and regional lymph nodes, into liver, lungs, spleen, heart and dermal lymphatics was observed. EM picture revealed no viral particles and MMTV-negativity was confirmed through MMTV LTR-specific RT-PCR. High expression of ER α, moderate to high expression of proliferating cell nuclear antigen (PCNA), moderate expression of vimentin and Cytokeratin 19 (K19) and low expression of p53 were observed in tumor sections, when compared with that of the normal mammary gland.

**Conclusion:**

Since 75% of human breast cancer were classified ER-positive and as our model mimics (in most of the characteristics, such as histopathology, metastasis, high estrogen levels) the ER-positive luminal epithelial-like human breast cancer, this model will be an attractive tool to understand the biology of estrogen-dependant breast cancer in women. To our knowledge, this is the first report of a spontaneous mammary model displaying regional lymph node involvement with both hematogenous and lymphatic spread to liver, lung, heart, spleen and lymph nodes.

## Background

Breast cancer is the most common malignancy in women and the mortality rate has been continuously increasing over the past 30 years. Based on ER level, about 70% of human breast cancers are phenotyped as ER-positive and others as ER-negative. Recently, micro-array studies have corroborated that majority (65%) of breast tumors are 'luminal epithelial-like/ER-positive' subtype, which express high levels of ERα and genes regulated by estrogen [1]

Mouse models are excellent tools to understand the natural biology of breast cancer. Since human breast cancers are clustered into several phenotypes (subtypes) based on grade, molecular-markers and micro-array studies, a good animal model for a subtype is one which mimics most of the subtype characteristics – morphology, molecular markers, metastatic pattern, grade, hormone-dependency, parity/pregnancy-status etc. [2,3]. Mouse tumors show a hematogenous spread almost exclusively to the lung, in contrast to human tumors, which show regional lymph node involvement with preferential spread to bone, brain, liver and heart. Also in mice, ER-positive and hormone-dependant mammary tumor is rare, where as this tumor subtype is found in majority(70%) of the human breast cancers [2,4] Recently, though there is a continuous arrival of new GEM models, it seems difficult to develop a 'high' similar mouse model of ER-positive and high-estrogen human breast cancer. [3,4].

We here report the development of ER-positive high-estrogen mammary tumor animal model from a spontaneously mutated NIH nude heterozygous female mice and the characterization based on histological, ultra structural, cellular and molecular approaches. At Present, there are almost no spontaneous or induced or genetically engineered mouse model available to study both hematogenous and regional lymph node dissemination with involvement of liver, spleen, heart and lungs even though it is the most frequent mode of dissemination for human breast cancer. To our knowledge, this is the first report of a mouse model showing metastasis both through hematogenous and lymphatic route.

## Methods

### Selection and propagation of tumor mice

In NIH nude mouse colony at animal house, Centre for Cellular and Molecular Biology (CCMB), we detected mammary tumors in one of the heterozygous breeding females. The tumor mouse was used as founder and continuous brother-sister mating with pedigree expansion system was followed to develop a medium-size heterozygous breeding colony showing high incidence of mammary tumors. F1 generation of brother sister mating produced offspring of 1/4 nu/nu (homozygous nude) ; 2/4 +/nu (heterozygous) and 1/4 +/+ mice. Wild type (+/+) and nu/nu mice does not show tumor. (0%) in their entire life span.

The animals were housed in accordance with the guidelines for care and use of animals in scientific research (Indian National Science Academy, New Delhi, India) in a CPCSEA (Committee for the purpose of control and supervision of experiment on animals) registered animal facility. The animals were maintained in Cabin type isolators at standard environmental condition (Temperature 22 – 25°C, Humidity 40 – 70%) with 12:12 dark/light photoperiod. No precise quantitative guidelines such as the acceptable upper limit of tumor burden was available, since the adverse effects on the host depend on the biology of the tumor, the site and mode of growth. But, we euthanized the mice before the size of tumor reached 10% of the animal's body weight.

### Tumor cytology & microbiology

Fine needle aspiration biopsy (FNAC) was taken from tumors and smears were prepared. The animals were euthanized and impression smears were made from the excised tumors. The Smears were air-dried, methanol-fixed, Giemsa-stained, and the images were captured under Zeiss axioplan microscope. 50 μl of tumor aspirate was transferred to 5 ml of nutrient broth (Himedia Laboratories, India) and the samples were kept at 37°C over night. Samples showing turbidity were streaked in Nutrient agar plates (Himedia Laboratories, India) and incubated at 37°C overnight. The colonies were stained with Gram stain and also streaked into blood agar and Mannitol salt agar plates (37°C). When colonies appeared, coagulase test was done.

### Hematological and biochemical assays

Body weight and tumor size were measured during initial stage of tumor progression, and at the time of sacrifice of animals to all the tumor-bearing animals (26 numbers) and normal animals (16 number). Complete blood picture [(Hemoglobin (Hb), Packed cell volume (PCV), Erythrocyte sediment rate (ESR), Red blood cells (RBC), White blood cells (WBC)] and differential leukocyte count were observed in all the tumor and normal animals as per standard procedure. Serum samples were analyzed for total protein, SGOT and SGPT by standard method using autoanalyser (autoanalyser2004 Bayer,). Serum estrogen and progesterone levels were measured by electrochemiluminescence immunoassay (Elecsys 1010/2010 and modular analytics E170 immunoassay analyzers, Roche Diagnostics, USA). Blood glucose levels were estimated by using digital AccuCheck sensor (Roche Diagnostics, USA).

### Histopathological analysis

Complete gross and histopathological evaluations were done. After euthanasia, mammary tumors and all organs were collected in 10% buffered Formalin (Liver, lungs, kidneys, heart, spleen, brain, pancreas, lymph node, bone, salivary gland, adrenals, small and large intestine, uterus, ovary, cervix and urinary bladder). Fixed and paraffin embedded tissues were cut at 5 μm thickness, stained with haematoxylin and eosin following standard procedure and examined under light microscope.

### Electron microcopy

Tumor tissues were fixed in 10% glutaraldehyde and then with 1% osmium tetraoxide in 0.1 M phosphate buffer, dehydrated in graded solutions of ethanol and embedded in LX – 112 (Fullium). Thin sections were stained with 1% Azur II plus 1% methylene blue in water for 3–5 min and then examined under light microscope. Ultramicrotome sections, contrasted with uranyl acetate and lead citrate were examined and photographed by transmission electron microscope (JEM-3100F TEM, JEOL Ltd, Japan). The sections were screened for MMTV and tumor cell morphology.

### Immunoflouresence assay using confocal microscopy

The level of expression of ER α, K18, K19, Vimentin, PCNA, p53, integrin α and Wnt-1, p63 were checked using immunofluorescence assay. Briefly, frozen section (16 μm) of tumor samples and normal mammary gland (control) were fixed with acetone for 20 minutes followed by permeabilization with 0.5% (v/v) Triton × 100 in PBS for 10 minutes at room temperature. After blocking with 5% horse serum in PBS for 1 hour, the sections were incubated with primary antibody for 1 hour and then incubated with FITC conjugated secondary antibody for 1 hour at room temperature. To reduce autofluorescence, the sections were treated with CuSO_4 _(10 mM) in ammonium acetate buffer (50 mM CH_3_COONH_4_, pH 5.5) for 30 minutes. The sections were counterstained with propidium iodide (PI) for 5 minutes and mounted in vector shield (Vector laboratories). The normal and tumor sections treated as above, but without primary antibody, served as negative controls. Lung and liver of tumor animals were also screened for the expression of ER α. The primary antibodies used were mouse monoclonal antibodies to ER α, (Catalog number – SC787; Santa Cruz Biotechnology, USA), Cytokeratins K18, K19, Vimentin, PCNA, p53, integrin α 5 and Wnt-1 (Santa Cruz Biotechnology, USA) and p63 (Chemicon International). Confocal laser scanning immunofluorescence microscopy (CLSM) was carried out using a Zeiss LSM 510 META confocal microscope. Image analysis was done using LSM510 META software (Carl Zeiss) and images were assembled using adobe Photoshop 7.0.

### RT-PCR with MMTV specific primers

RNA were isolated from tumor of NIH heterozygous mice and mammary gland of normal NIH nude heterozygous mice, normal inbred c57BL/6 J and c3H/HeJ mice using TRIZOL Kit (GIBCO-BRL) according to the instruction provided by the supplier. Each RNA sample was subjected to RT-PCR with MMTV LTR-specific primers FPC1 5'GACATGAAACAACAGGTACATGA3' and RP 5'GGACTGTTGCAAGTTTACTC 3' based on a standard procedure [5]. Genomic DNA were isolated from tumor mice and inbred mice (c57BL/6 j and c3H/he J mice) tail as positive control using phenol chloroform method. Genomic DNA were amplified with MMTV LTR specific primer. Addition to this, CDNA from tumor was amplified with beta actin primer (βactin432F 5' GCG TGA CAT CAA GGA GAA GC 3' and βactin432R 5' TGG AAG GTG GAC AGG GAG GC 3') as a positive control.

### Hormone responsiveness of mammary tumor

We selected six tumor bearing animals for ovarioectomy study and among this animal number 42, 46 and 47 as treated group and rest of the three as positive control. The animal was anesthetized using Ketamine (40 mg/kg body weight) and Xylazine (10 mg/kg body weight) through intraperitoneal injection and Overectomy was done in bilateral as per standard guideline for rodent surgery. Tumor volume and pre operative serum estrogen and progesterone were measured by chemiluminescence's immunoassay. Serum estrogen, progesterone levels and tumor volume were also checked for every seventh day from the day of removal of ovary to 28 days from all the six animals. Tumor Volume was calculated by using standard formula made earlier [V = 0.4(ab^2^); a – length of the tumor; b – height and width of the tumor] [6].

## Results

### Animals

**Figure 1:**
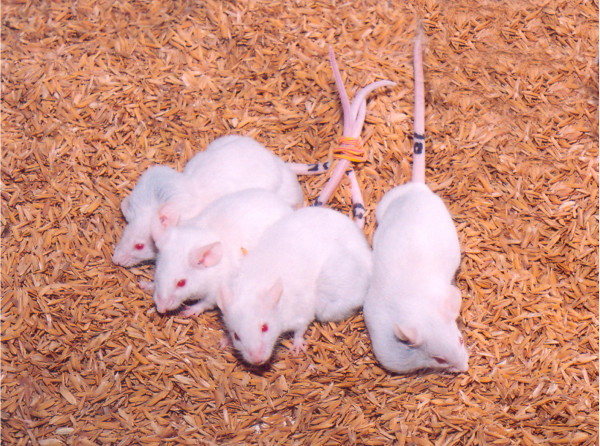
Biparous heterozygous NIH nude females showing mammary tumors.

Mammary tumors were observed only in female breeding-mice, mostly after delivering the second litter. The females which were not mated did not develop tumors in its life span. Among 42 females bred, 26 females developed mammary adenocarcinoma (incidence rate 62%). The age of occurrence of tumor was about 7 months (range, 3.5 to 12 months). The tumors were unilateral i.e., developed either in right or left side mammary glands and were observed only in 2^nd ^or 5^th ^or both mammary glands (Fig. 1). The tumor grew rapidly and reached 10% of the body weight in 3–5 weeks. At the time of euthanasia, the average body weight was about 33 gm and the tumor-size was about 3 cm × 3 cm × 2 cm (l × w × h).

### Tumor cytology & microbiology

**Figure 2:**
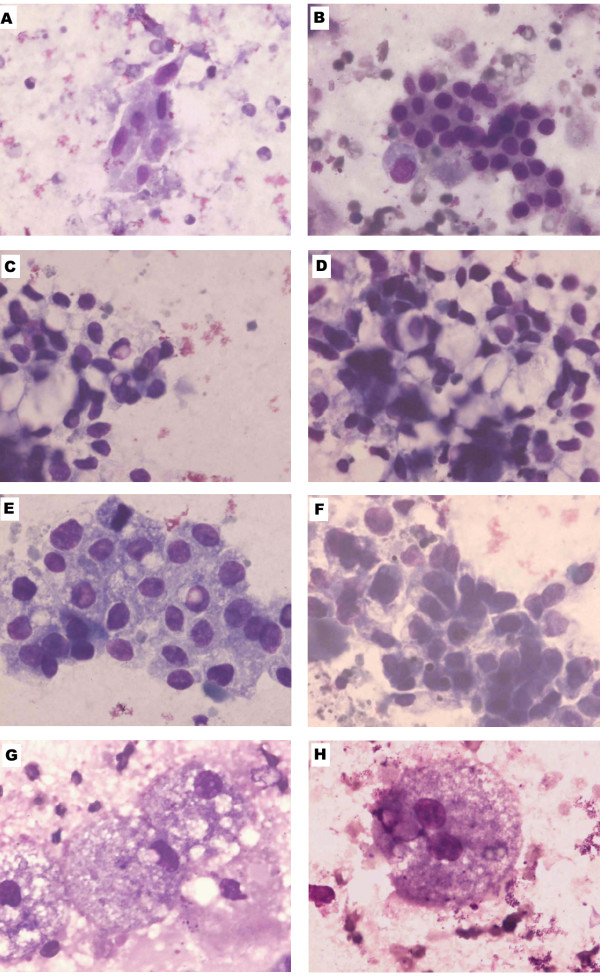
**Fine needle aspiration Cytology**. A. A small cluster of spindle and round cells with pleomorphic nuclei. B. A medium sized cluster with loosely cohesive round cells with hyper chromatic nucleus. C&D. A Large cluster with very loosely cohesive signet ring cells with eccentric, hyperchromatic, pleomorphic nuclei. E&F A large cluster with loosely cohesive round to polygonal foamy cells with hyperchromatic nuclei. G. Giant-sized foamy cells with eccentric nuclei. H. Giant binucleated foamy cell. Magnification (Giemsa, × 100).

Smears showed low to moderate cellularity. Small to large clusters of loosely cohesive cells with hyper chromatic nucleus were found. Tumor cells were pleomorphic. The cells were spindle, round, polygonal or signet ring shaped; small to giant sized with normal or foamy cytoplasm and eccentric or centrally placed nucleus. (Fig. 2). Golden-pigmented colonies were seen in mannitol salt agar. Smears showed gram-positive grape-like clusters of organisms. Colonies were coagulase positive suggestive of *Staphylococcus aureus*.

### Hematological and biochemical assay

Mean ± SE values of various hematological and biochemical parameters were displayed in Table 1. While comparing with normal control, the level of RBC was low and the levels of neutrophils, estrogen, SGPT and SGOT were high.

**Table 1:** Significant increase in estrogen and liver enzyme

Parameters	Tumor mice (Mean ± SE)	Normal Value (Range)
Age of Tumor Incidence	7.2 ± 0.4 months	-
Body Weight	33.5 ± 0.68 g	29.6 ± 0.68 g
Glucose	111 ± 1.9 mg/dl	126 ± 1.9 g/dl
Hemoglobin	13 ± 0.2 g/dl	11.6 g ± 0.12 g/dl
RBC	4.7 ± 0.08 × 10^6^	8.6 ± 0.14 × 10^6^
WBC	9650 ± 93/mm^3^	3250 ± 12/mm^3^
Neutrophils	60.5 ± 0.4%	32 ± 0.28%
Lymphocytes	37.4 ± 0.3%	48 ± 0.16%
Eosinophils	1.8 ± 0.17%	1.6 ± 0.13%
Estrogen	15.1 ± 0.22 pg/ml	1.2 ± 0.13 pg/ml
Progesterone	0.13 ± 0.006 ng/ml	0.1 ± 0.04 ng/ml
SGPT	58.2 ± 1.2 IU/L	23 ± 2.6 IU/L
SGOT	70.3 ± 0.66 IU/L	28 ± 1.2 IU/L
Albumin	3.34 ± 0.04 g/dl	3.2 ± 0.07 g/dl
Total Protein	6.65 ± 0.04 g/dl	5.2 ± 0.12 g/dl

### Histopathological analysis

**Figure 3:**
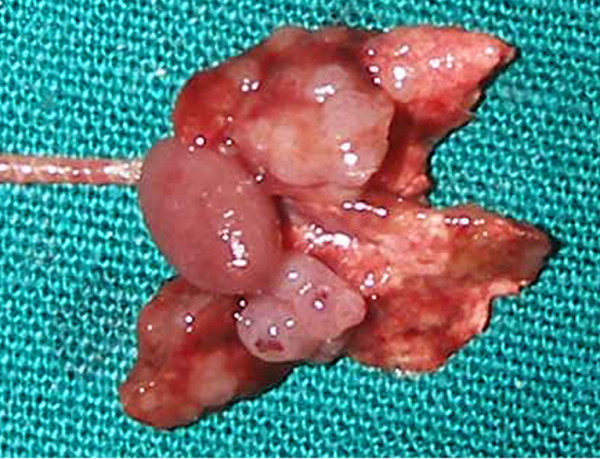
Metastasis in Lungs.

Neoplastic mass, while it reaching 1/10^th ^body weight, appeared as multi-lobes with ulceration on the skin surface. On palpation, fluid thrills were observed in each lobe. Exteriorized tumor mass revealed multi-lobes, each lobe had multiple solid nodules enclosing a central necrosed tissue with foul smelling inflammatory exudate and clotted blood. Multiple pinkish metastatic nodules were observed in all the lobes of lungs. (Fig 3) lung nodules varied from 0.7 × 0. 5 × 0.3 cm to 0.3 × 0.25 × 0.15 cm. Splenomegaly with multiple white colored nodules popping out from inner splenic parenchyma were observed. Liver and peripheral lymph nodes were enlarged in all the animals.

**Figure 4:**
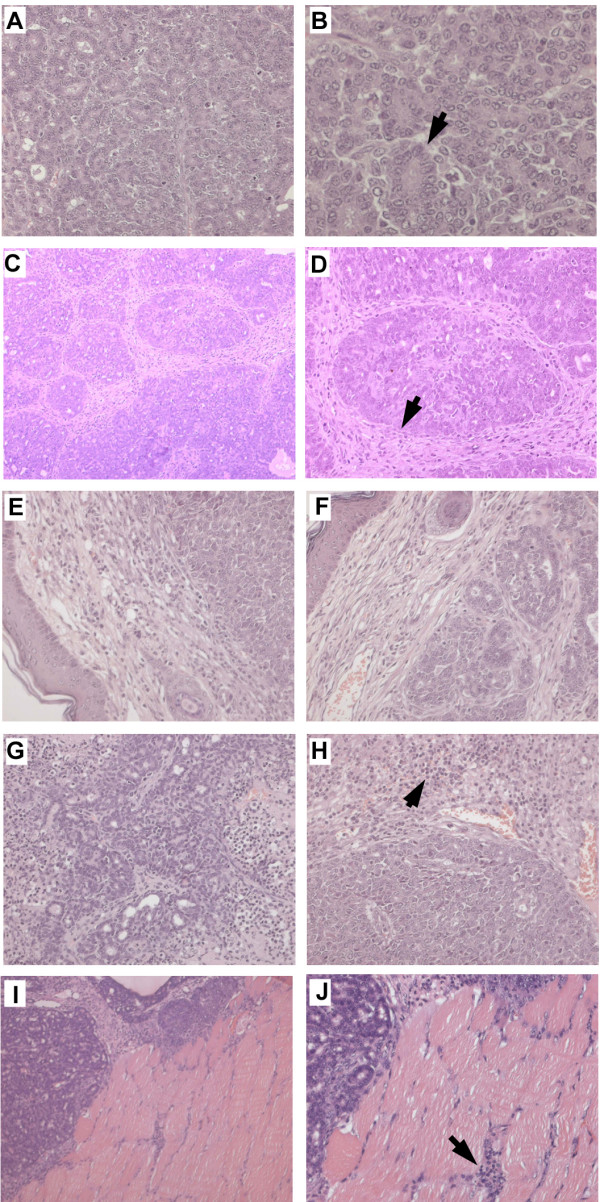
**Tumor morphology**. A&B. Luminal/tubular epithelial-like morphology. Tubules lined by single or multiple layers of pleomorphic epithelial cells. (arrow) (A-H&E, ×200 ; B- H&E, × 400). C. Lobules separated by stroma (arrow). H&E, ×100 D.A lobule surrounded by stoma (arrow). H&E, ×200. E&F. Tumor mass pushing the adjacent dermis, H&E, ×200.G&H. Neutrophils invading the necrosed areas in the tumor mass (arrow). H&E, ×200. I&J. Neoplastic epithelial cells with hyperchromatic nucleus invading the underlying muscle layers (arrow)(I-H&E, ×100; J- H&E, × 200).

Histologically, the expansile tumor mass pushed through the overlying dermis and was found infiltrating the muscle layer underlying the mammary gland. The tumor mass resembled luminal/tubular epithelial-like morphology with well differentiated (Grade 1) lobes. Each lobe had lobules showing acinar or cystic or combination of acinar, cystic and inflammatory patterns. (Fig. 4). The tubules were lined by single or multiple layers of pleomorphic epithelial cells. Some region of the tubules were filled with amorphous inflammatory exudate appeared as cystic pattern. The neoplastic epithelial cell had indistinct borders and contained scant to moderate amount of cytoplasm with pleomorphic nucleus with mitotic figures. Multiple area of necrosis with infiltration of neutrophils appeared in between and within lobules.

**Figure 5:**
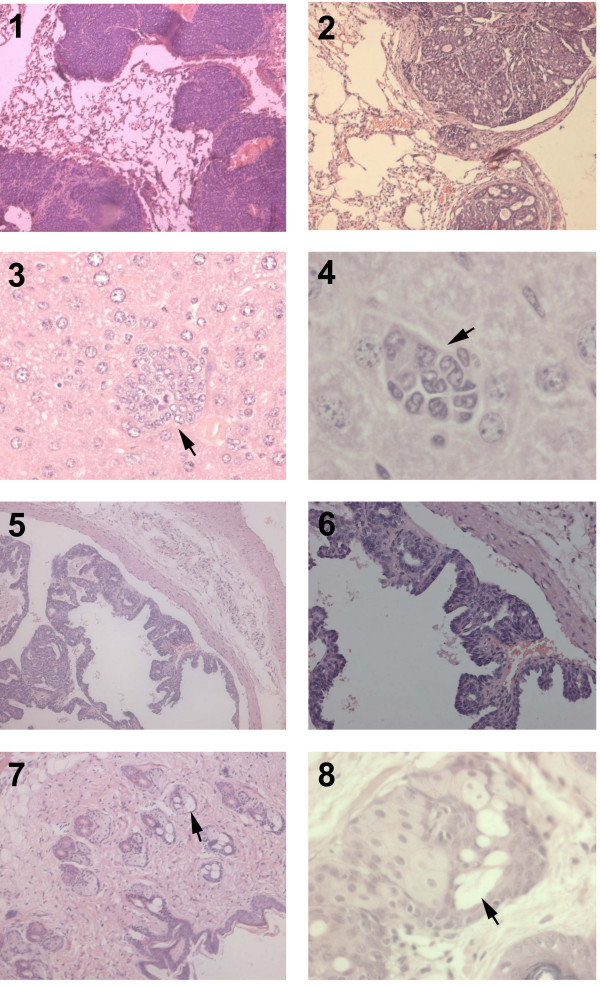
**Tumor Metastasis**. 1 & 2. Metastatic foci in lungs showing luminal epithelial-like morphology as in primary with well differentiated secretory glands (1-H&E, ×100 ; 2- H&E, × 200).3 & 4. Metastatic foci in liver. (arrow)- 3 -H&E, ×200 ; 4- H&E, × 400).5 & 6. Metastatic nodules growing on the vascular endothelium of aorta (5-H&E, ×100 ; 6- H&E, × 200). 7 & 8 Sebaceous glands showing pleomorphism, moderate differentiation and loss of polarity (arrow) (7-H&E, ×200 ; 8- H&E, × 200).

Neoplastic tubular epithelial cells metastasized into lungs, liver, spleen, lymph node, heart, dermal lymphatic vessels and sebaceous gland (Fig 5). No metastatic foci in bone, salivary glands, adrenal, kidney, brain, intestines, and pancreas were detected. Entire alveolar region of lungs were occupied by multiple lobes of tubular neoplastic cells. The morphology of lung tumors was similar to that of mammary tumors. Multiple foci of tumor emboli consisting of pleomorphic neoplastic cells spreading through hepatic sinusoids were found in liver. Large sized pleomorphic neoplastic cells were found in splenic and lymphatic follicles. Nodular-type tumors consisting of pleomorphic tubular epithelial cells were found attached to inner wall of aorta. Pleomorphism, moderate differentiation and loss of polarity in sebaceous glands were suggestive of metastatic invasion. Few necrotic foci and sparse distribution of individual pleomorphic cells in cerebral hemisphere were observed.

### Ultra structure

**Figure 6:**
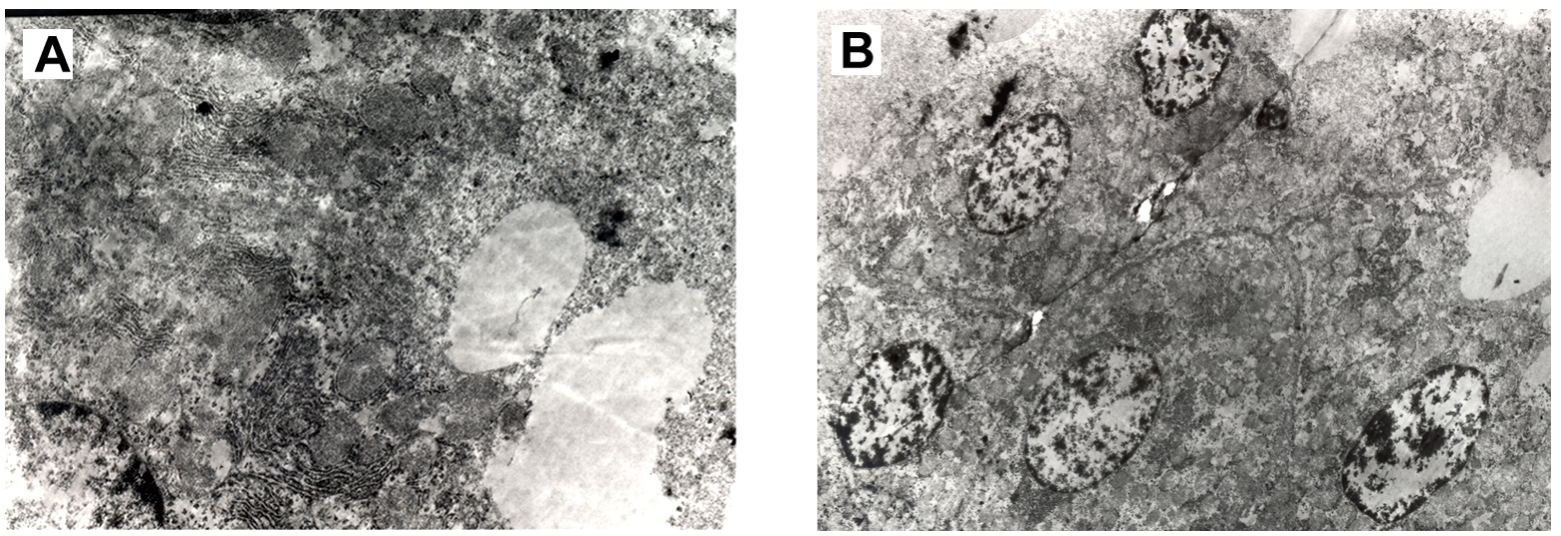
**Electron microscopy of tumor section**. A&B : High number of endoplasmic reticulam in the tumor epithelial cells. No viral-like particles detected. Magnification × 6000.

Electron microscopic picture revealed round to oval shaped dark epithelial cells Large numbers of endoplasmic reticulum and secretory granules were observed in the cells (Fig 6). Increase in endoplasmic reticulum indicated higher metabolic activity, protein and lipid synthesis in the cells. None of the sections showed myoepithelial cells and MMTV virus particles. Euchromatin was predominantly observed inside the nuclear membrane. The predominance of euchromatin is attributable to the high percentage of cells in DNA synthesis phase (S phase) and this is usually seen in cancerous cells.

### Confocal microscopy

**Figure 7:**
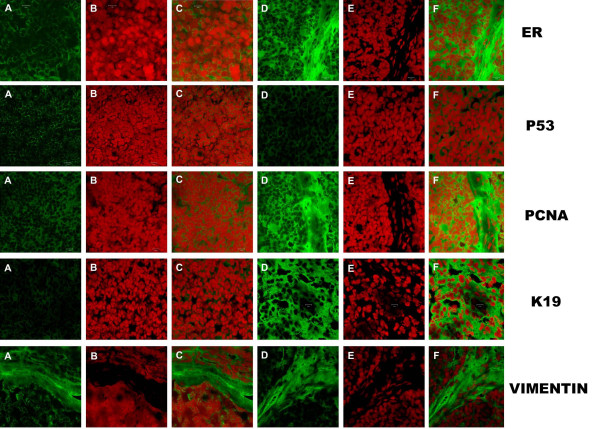
**Immunoflouresence assay**: Intensity of Greenfluorescence indicates the expression level of the correspondingmolecular markers. A, B, C – Normal mammary gland section of respectivemarker; D, E, F – Tumor section of respective marker. Magnification ×1000. A – FITC alone; B – PI alone; C – Dual (FITC & PI). D -FITC alone; E – PI alone; F – Dual (FITC & PI). ER: Higher expression inneoplastic epithelial cells (F) than normal controls(C). P53: Lowerexpression in neoplastic epithelial cells (F) than normal controls C. PCNA: Moderate to higher expression in neoplastic epithelial cells (F) than normal controls(C). K19: Moderate expression in neoplastic epithelial cells (F) than normal controls(C). Vimentin: High expression of vimentin in the stromal cells (F) than normal controls (C).

High expression level of ER α was found in neoplastic epithelial cells of all the tumor sections when compared with normal mammary gland controls. Expression of K18 and K 19 were high in neoplastic epithelial cells. Higher expression of vimentin in stromal cells and also polymorph nuclear cells and mononuclear cells, which were frequently found within the lobes of tumor strongly, reacted with vimentin antibodies. Low expression of p53, mild to moderate expression of p63 and moderate to high expression of PCNA in neoplastic epithelial cells were seen. (Fig 7). Wnt-1 and integrin α 5 were not expressed. No immunofluorescence was observed in the negative controls.

### MMTV LTR-specific RT-PCR

**Figure 8:**
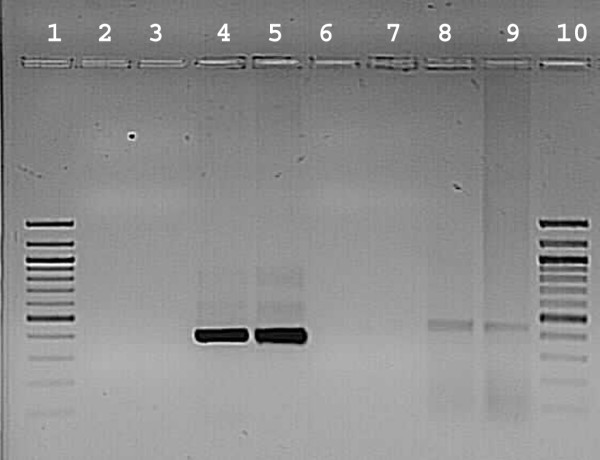
**MMTV screening with RT-PCR**. Lane 1 & 10 -100 bp laddar; 2 & 3 – CDNA from tumor sample; 4 & 5 Genomic DNA of C57 and C3H mice (426 bp); 6 & 7 – RNA from c57 and c3H mice; 8 & 9 – CDNA from tumor sample with β actin primers (432 bp).

PCR using MMTV LTR-Specific primers (FPC1& RP) with cDNA template from tumor tissue, RNA from inbred mice did not show any amplification, but genomic DNA of inbred mice c57BL/6 J and c3H (positive control) showed amplification of 426 bp. PCR using βactin primers with CDNA template from tumor tissue showed amplification of 432 bp. (Fig 8). These results indicate MMTV negativity in our animal model.

### Hormone responsiveness of mammary tumor

Detailed hormone responsiveness has been displayed in Table 2 &3. Estrogen and progesterone and tumor size were reduced from seventh days onwards. No serum estrogen and progesterone were observed on and after 14 days. Tumor size was gradually reduced from seventh days onwards and on 28 th day tumor size was reduced to more than half the size of the tumor on day 1.

**Table 2:** Hormone responsiveness in tumor mice

	Serum Estrogen pg/ml in days										Serum Progesterone ng/ml in days									
	0		7		14		21		28		0		7		14		21		28	
	
An. No O/P	O	P	O	P	O	P	O	P	O	P	O	P	O	P	O	P	O	P	O	P
42/01	12.2	1.2	0.5	0.9	-	0.9	-	1.2	-	1.2	0.1	0.08	0.03	0.1	-	0.07	-	0.08	-	0.08
46/02	16.5	0.9	0.3	0.8	-	1.3	-	1.2	-	0.8	0.2	0.09	0.02	0.12	-	0.08	-	0.09	-	0.09
47/03	14.7	0.7	0.4	0.7	-	0.8	-	1.2	-	1.2	0.1	0.08	0.06	0.08	-	1.2	-	0.09	-	1.1

O – Overectomized tumor animals; P – Positive control of tumor animals

**Table 3:** Hormone responsiveness in tumor mice

	Tumor volume in ml per days									
	0		7		14		21		28	
	
An. No O/P	O	P	O	P	O	P	O	P	O	P
42/01	7.7	6.8	6.1	7.0	3.5	7.3	1.3	7.3	0.25	7.8
46/02	3.8	4.2	4.1	4.6	1.8	5.2	1.0	5.8	0.4	5.8
47/03	2.4	4.8	0.9	4.8	0.8	5.2	0.4	5.6	0.2	5.7

O – Overectomized tumor animals; P – Positive control tumor animals.

## Discussion

We have developed a spontaneous mammary tumor model with high level of ER expression along with high levels of serum estrogen. The model displayed metastasis, through both hematogenous and lymphatic route, into regional lymph nodes, liver, lung, heart, spleen and lymph nodes. The tumors predominantly had luminal/tubular epithelial-like morphology. All the above characteristics make the model resemble closely to the ER-positive, luminal epithelial- like subtype of human breast cancer.

High levels of endogenous sex steroid hormones are associated with increased risks of breast cancer in postmenopausal women and levels of circulating estrogens and androgens may be important in the etiology of premenopausal breast cancer [7]. Most of the study disseminated the information that steroid and peptide hormones have a considerable effect on the initiation of mammary tumorigenesis [8]. Rat models are found to be better than mouse models for study of estrogen signaling and tumorigenesis in vivo, since they show high frequency of ER-positive lesions [8]. Ironically, the techniques for targeted manipulation of rat genome is in the developmental stage [3] and rats predominantly show fibroadenoma rather than adenocarcinoma mammary tumors [9]. In contrast to rat, mouse predominantly show adeno carcinoma but hormone – independent mammary tumors [4].

Majority of human breast cancers (75%) are ER positive, [10] but vast majority of mammary lesions in GEM are ER – negative. To our knowledge, few GEM ER + animal model have been made [11-15] in the recent past. But, most GEM does not precisely recapitulate steroid receptor signaling during neoplastic transformation.

In chemically induced tumor model, medroxyprogesterone produced mammary adenocarcinoma in balb/c mice possess estrogen and/or progesterone, PRL, and EGF receptors was reported earlier [16,17]. Traditional models and recent data from genetically engineered mouse (GEM) models suggest many similarities between mammary cancers of mice and human at gene/pathway-level, but important differences in the biology of metastasis and the preponderance of ER- positivity and hormone-dependency [3,4]. Presently xenograft models are used in preclinical studies. But these models are immunocompromised and are unable to express the steroid hormones at the near-physiological levels. This makes the model inappropriate for studies on immunotherapy, anti-hormonal therapy. Also, Xenograft model needs human stromal cells to be co-transplanted with human epithelial cells to provide local growth factors needed for epithelial tumor growth. Though the human epithelial cells would metastasize lung or bone, they will only interact with murine stroma. In therapeutic studies, targeting stroma of metastasizing breast cancer would be inadequate in xenografts models using tissue-recombination approaches. In this context, our immunocompetant models (heterozygous nude females) showing high serum estrogen mimicking their human counterparts should be useful for immunotherapy and anti-hormonal therapy studies.

MMTV was incriminated for most of the mammary tumors in mice. MMTV exists as both an endogenous and exogenous virus transmitted to pups via the germ line and breast milk. All inbred strain of mice as well as some wild mice contain multiple copies of MMTV proviral integrants, although most of them fail to produce infectious MMTV particle. (5). The virus does not contain oncogene but inserts near known sets of proto-oncogenes resulting in up regulation and tumorigenesis [18]. MMTV infection activates the notch, Wnt and FGF family genes. Wnt -1 was discovered as a gene frequently activated in mammary tumor arising in mice infected with MMTV [19]. In our model, electron microscopic picture of mammary tumor revealed no viral like particles, immunofluorescence assay showed that expression level of Wnt – 1 was not different from that of normal controls. The RT-PCR results further corroborates MMTV negativity in our models. Previous studies show that correlation between activation of Wnt-1 and ER positivity could not be established in mouse models. For example, estrogen receptor positivity in mammary tumors of wnt – 1 transgenic mice is influenced by collaborating oncogenic mutations rather than wnt-1 per se [9]. Mouse mammary tumor virus-Wnt-1 transgene also induced mammary gland hyperplasia and tumorigenesis in mice lacking estrogen receptor-alpha, though it is not known whether ER signaling is necessary for survival of the ER + tumors that develop in Wnt-1 TG mice [9]. Also, no positive correlation between MMTV viral sequence and estrogen receptor positivity in human breast cancer was reported earlier [20].

Aspiration biopsy, tumor cells were arranged in clusters and dissociation with spindle, binucleated, round to polyhedral clusters and foamy clusters of signet ring shaped cells. The same kind of cytological observations were reported earlier with FNAC [21] in human breast cancer. Microbiological examination confirmed the tumor mass infected with Staphylococcus aureus. The presence of bacteria might be due to secondary invasion.

High estrogen levels correlated with higher expression pattern of ER and also increased levels of SGPT, SGOT indicative of liver damage due to metastasis or tumor emboli. Higher the levels of neutrophils support the inflammation and necrosis between the lobes of tumor mass. Overectomy experiment indicates that, our animal model has responded to ovarian hormone and majority of ER + tumors respond to antiestrogen therapies despite their high or low levels of ER expression [22]. We could not interpret the hormone responsiveness based on three ovarioectomy mice. We require lot of further experiments (antiestrogenic therapy with tamoxifen, replacing estrogen levels with pellet) with more number of animals.

Our model shows both hematogenous and regional lymph node dissemination with involvement of liver, spleen, heart and lungs. But, human breast cancers commonly metastasize into regional lymph nodes. The reason for metastasis into regional lymphnodes in this mouse model is still unclear. This might be due to aggressive growth or high cell number due to increased tumor burden. Mammary tumor manipulation induced dissemination to the axillary nodes and increased up to 6-fold the number of metastatic lung nodules [23]. The average percentages of lymph node metastasis in mouse inoculated with mouse hepatocellular carcinoma Hca-F cells increased in an almost proportional fashion with the number of cells implanted subcutaneously [24]. More future studies are needed to explain the regional lymph node involvement in mouse models since there are basic differences between human and mouse mammary tissue in epithelium-stroma microenvironment.

## Conclusion

Metastasis rather than primary tumor are responsible for most breast cancer related to deaths in human. Since our model mimics most of the characteristics of the major(70%) human breast cancer subtype(Luminal epithelial-like/ER positive) and shows metastasis into regional lymph nodes and distant organs, we believe this has a major application potential in the human breast cancer studies.

## Competing interests

The author(s) declare that they have no competing interests.

## Authors' contributions

KSP & KK carried out MMTV screening and assisting manuscript preparation. RSN -screen the microbes. JJ – carried out animal management, tumor measurement, blood collection etc. GS – Histopathology & frozen section slide preparation. PN & RV – conceived of study, and participated in its design and co ordination. RN – analyzed the immunoflouresence assay using confocal microscopy. SS – carried out electron microscopic study. All authors have read and approved the final manuscript.

## Pre-publication history

The pre-publication history for this paper can be accessed here:


